# Combination of Antidepressant and Alcohol Intake as a Potential Risk Factor for Rhabdomyolysis

**Published:** 2018-09

**Authors:** Dong Jun SUNG, Miyea LEE, Ji-Kang PARK, Hyun-Jung PARK

**Affiliations:** 1. Division of Sport and Health Science, College of Biomedical and Health Science, Konkuk University, Chungju, Chungbuk 27478, Korea; 2. Dept. of Nursing Science, Chungbuk Health and Science University, Cheongju, Chungbuk 28150, Korea; 3. Dept. of Orthopedics, Chungbuk National University Hospital, Cheongju, Chungbuk 28644, Korea; 4. Dept. of Nursing, College of Social Services, Pyeongtaek University, Pyeongtaek, Gyeonggi, 17869, Korea

## Dear Editor-in-Chief

Rhabdomyolysis (RML) is generally recognized as a serious condition due to sarcolemma damage with release of creatine kinase (CK) and myoglobin (Mb). Causes of rhabdomyolysis are variable, including mechanical damage, infection, genotype, and intense exercise (1). RML is caused by a single or mixed ingestion of some drugs and foods (2), especially antidepressant drugs and alcohol.

Taking selective serotonin reuptake inhibitors (SSRIs) after eccentric exercise could aggravate rhabdomyolysis (3). Administration of low-dose venlafaxine, a serotonin-norepinephrine reuptake inhibitor (SNRI), has been associated with severe RML (4). In addition, serotonin syndrome induced by administration of SSRIs and SNRIs can lead to RML and acute renal failure (5).

Although the precise mechanism by which antidepressant drugs lead to RML remains unclear, serotonin syndrome and increased serotonin transmission in the central nervous system are associated with autonomic dysfunction and a spectrum of neuromuscular and cognitive symptoms (6). Serotonin-induced RML after medication with SSRI or SNRI is also associated with CNS-mediated muscle rigidity, tremor, and activity (3, 6).

Alcohol is mainly metabolized into acetaldehyde. The relevance of alcohol to RML is probably due to the fact that acetaldehyde can alter Ca^2+^-release channel gating and muscle contraction in a dose-dependent manner (7). Moreover, myopathy in skeletal muscle can be induced by chronic alcohol consumption (8). Intracellular mechanisms that lead to RML are predominantly affected by an increase in intracellular Ca^2+^ concentration (9). Therefore, alcohol might be a potential risk factor for RML through increasing intracellular Ca^2+^ concentration and inducing myopathy. RML can also be caused by alcohol and drug use in combination. For example, RML and acute renal failure are caused by alcohol consumption and overdose of diphenhydramine, an antihistamine drug (10). However, RML induced by a combination of antidepressant drug and alcohol intake has not been reported yet. Here, we report a case of RML induced by a combination of antidepressant drug and alcohol intake. A 40-year-old man (height=175 cm, weight=67.2 kg) with depression was on antidepressant treatment with desvenlafaxine (50 mg/day), an SNRI class drug. He had no illness or family history other than depression. He did not have any physical activity that could cause RML or muscle soreness. Before RML, he drank about two bottles of Soju (17.8% of alcohol). The patient’s pulmonary and circulatory systems were normal when he was admitted to the emergency room. However, some markers in hematological lab test, biochemical examinations, and urinalysis revealed abnormal findings associated with RML.

His levels of aspartate transaminase (AST) and alanine transaminase (ALT) were 1573 IU/L (reference: 0–40 IU/L) and 562 IU/L (reference: 0–40 IU/L), respectively. His level of C-reactive protein, a blood inflammatory marker, was 33.68 mg/dl (reference: lower than 0.3 mg/dl). His levels of CK and CK-MB, markers of RML, were measured to be 36100 U/L (reference: lower than 190 U/L in male) and 139.7 ng/ml (reference: lower than 4.94 ng/ml), respectively. The patient’s level of lactate dehydrogenase, a marker of myocyte damage, was 3170 IU/L (reference: 240–480 IU/L). Based on these results, the patient was diagnosed with RML. He had no complications such as acute renal failure or compartment syndrome.

Antidepressant medication, especially SNRI, is a potential risk factor for RML if alcohol is consumed. Therefore, patients who are taking antidepressants should avoid alcohol intake. Physicians should be aware of the risk that combining antidepressant drug and alcohol can lead to non-traumatic RML. Further study is needed to understand the mechanism by which a combination of antidepressants and alcohol leads to RML.
